# Pharmacological blockade of cannabinoid receptor 2 signaling does not affect LPS/IFN-γ-induced microglial activation

**DOI:** 10.1038/s41598-023-37702-z

**Published:** 2023-07-10

**Authors:** Bolanle Fatimat Olabiyi, Anne-Caroline Schmoele, Eva Carolina Beins, Andreas Zimmer

**Affiliations:** 1 Institute of Molecular Psychiatry, Medical Faculty, University of Bonn, Bonn, Germany; 2 Institute of Human Genetics, University of Bonn, School of Medicine & University Hospital Bonn, Bonn, Germany

**Keywords:** Immunology, Neuroscience, Biomarkers, Pathogenesis

## Abstract

Cannabinoid receptor 2 (CB2) signaling modulates microglial responses to inflammatory stimuli. Our previous studies demonstrated that genetic deletion of CB2 inhibits microglial activation during inflammatory stimulation of toll-like receptors (TLRs) or in neurodegenerative conditions. However, we cannot exclude developmental effects of the constitutive CB2 knockout (CB2^−/−^), which could mediate compensatory outcomes in CB2^−/−^ mice. In the present study, we therefore tested whether acute pharmacological inhibition of CB2 receptor has a similar effect on microglial activation as in CB2^−/−^ in response to inflammatory stimulation. Our findings suggest that the CB2-specific antagonist SR144528 has little or no effect on LPS/IFN-γ-induced activation in primary microglia or organotypic hippocampal slice cultures at nanomolar concentrations. We show that SR144528 did not alter LPS/IFN-γ-mediated microglial cytokine secretion, Iba1 and CD68 staining intensity or morphology at 1 and 10 nM. Although SR144528 suppressed LPS/IFN-γ-induced microglial activation at 1 µM, this anti-inflammatory effect was not dependent on CB2 receptors and exceeded the Ki on CB2 receptors by more than a thousand-fold. Thus, SR144528 does not mimic the anti-inflammatory effects observed in the CB2^−/−^ microglia after LPS/IFN-γ stimulation. Therefore, we propose that the deletion of CB2 probably triggered an adaptive mechanism, making microglia less responsive to inflammatory stimulation.

## Introduction

Cannabinoid receptor 2 (CB2) is one of two G protein-coupled receptors of the endocannabinoid system, a complex neuromodulatory system expressed throughout the body^1^. CB2 receptors are primarily present on peripheral immune cells and microglia^2,3^, where they exhibit important immune-regulatory functions^4,5^. Numerous studies have employed both genetic and pharmacological approaches to study the role of CB2 signaling in neuroinflammatory conditions^6–9^. Both methods have advantages and disadvantages. Where constitutive or cell type-specific deletion of CB2 receptors can provide information about CB2 functions^10^, compensatory responses triggered by genetic manipulation may confound data interpretation. Pharmacological approaches, on the other hand, can provide information about the functional consequences of acute CB2 blockade^8,9^, but they are strongly dependent on the selectivity/specificity of the pharmacological compound. In this context, it is essential to note that many pharmacological studies used CB2 antagonists at doses that far exceeded the dose required for a full blockade of CB2 signaling^9,11,12^.

In our previous studies, we demonstrated that CB2 deletion (CB2^−/−^) in an APP/PS1 mouse model of Alzheimer's disease resulted in improved cognition and lower inflammatory responses^13^. Interestingly, the morphology of plaque-associated microglia in APP/PS1*CB2^−/−^ mice indicated that they were less activated compared to those of APP/PS1 control mice. To further address the underlying cell mechanism, we also studied the in vitro response of microglia isolated from CB2^−/−^ mice. Our results indicated that treatment of these microglia with lipopolysaccharide/interferon-gamma (LPS/IFN-γ) resulted in a decreased secretion of inflammatory cytokines and reduced surface expression of activation markers^6,14^. Using gene expression profiling, we next showed that CB2^−/−^ microglia have an attenuated toll-like receptor (TLR)-induced gene activation profile^14^. Thus, CB2 receptors seemed to be required for full activation of microglia during inflammatory stimulation.

These results were somewhat surprising because CB2 agonists also produce anti-inflammatory effects on microglia and macrophages in response to inflammatory stimuli. For example, JWH-015 and AM1241 suppressed inflammatory responses to IFN-γ or LPS/IFN-γ stimulation^7,15^. Moreover, JWH-133 increased the production of the anti-inflammatory cytokine IL-10 in TLR4-stimulated macrophages, and co-administration of the CB2 antagonist SR144528 blocked this effect^9^. In vivo, CB2 activation with JWH-133 similarly improved cognition in an AβPP/PS1 mouse model and lowered inflammatory cytokine expression and microglia reactivity^8^. Additionally, the CB2 agonist 0-1966 reduced the number of activated microglia and enhanced motor function in another model of traumatic brain injury^16^.

Considering the above-mentioned limitations, it is not surprising that genetic deletion and acute pharmacological inhibition can have different outcomes^17^. Our findings of reduced microglial activation in CB2^−/−^ mice raised the question of whether this effect was caused by the acute lack of CB2 signaling or was the result of compensatory mechanisms. To address this, we now analyzed the effect of acute treatment of LPS/IFN-γ-stimulated microglia with the potent selective CB2 antagonist/inverse agonist SR144528^18,19^. SR144528 is among the most commonly used antagonists, due to its high affinity and selectivity for CB2 receptors at subnanomolar concentrations^18^, with an inhibitory constant (Ki) of 0.6 nM^18^.

## Results

### Pharmacological blockade of CB2 signaling has no effect on LPS/IFN-γ-induced microglial cytokine secretion

**Figure 1:**
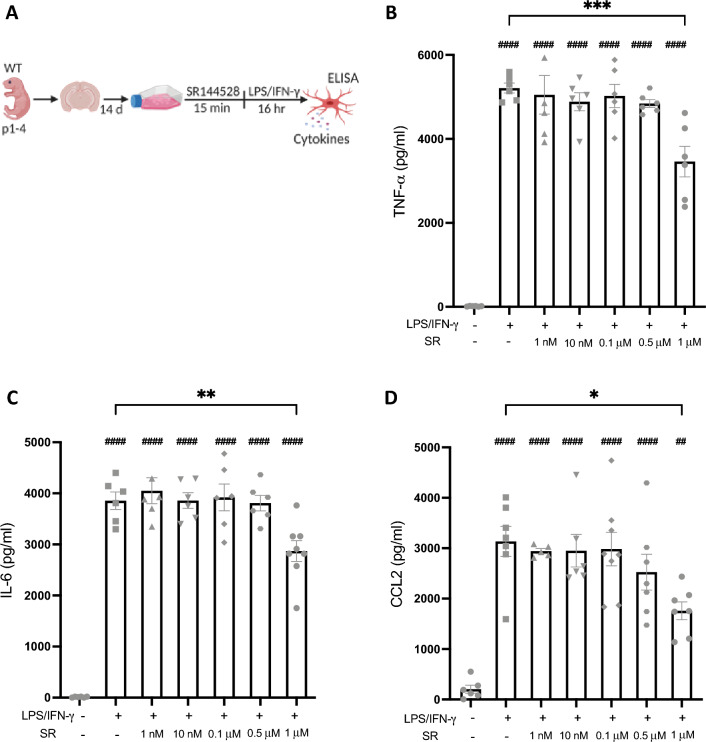
SR144528 reduced LPS/IFN-γ-mediated microglial cytokine/chemokine secretion only at high concentrations. (**A**) Experimental setup. Primary microglia were pre-treated with SR144528 at the indicated concentrations for 15 min, followed by LPS/IFN-γ stimulation for 16 h. (**B**–**D**) Levels of cytokines/chemokines were quantified in supernatants using ELISA. (**B**) TNF-α, (**C**) IL-6, and (**D**) CCL2. N = 5–8 samples/stimulation (representative data from three independent preparations). Data are presented as mean ± SD. One-way ANOVA followed by Tukey's multiple comparisons. #### *p* < 0.0001, ## *p* < 0.01 indicate significance to the untreated control group. Significant differences between LPS/IFN-γ vs. LPS/IFN-γ pre-treated with SR144528 are indicated with *** *p* < 0.001, ** *p* < 0.01, * *p* < 0.05.

To assess whether acute pharmacological inhibition of CB2 signaling has similar effects on LPS/IFN-γ-induced microglial activation as the constitutive deletion of CB2, we tested the effects of increasing concentrations of SR144528^18^ (1 nM–1 µM) on LPS/IFN-γ-induced cytokine secretion in wildtype (WT) primary microglia (Fig. 1). Stimulation with LPS/IFN-γ significantly increased the secretion of all cytokines tested (*p* < 0.0001). This response was not affected by pre-treatment with SR144528 at nanomolar concentrations, where the compound is CB2 selective. Only a concentration of 1 µM significantly reduced LPS/IFN-γ-induced secretion of TNF-α (*p* = 0.0008), IL-6 (*p* = 0.0089), and CCL2 (*p* = 0.0140). This concentration exceeds the Ki of SR144528 on the CB2 receptors by more than three orders of magnitude, suggesting that the observed effect could be CB2-independent.

### SR144528 (1 µM) reduces LPS/IFN-γ-induced microglial inflammatory cytokine secretion independent of the CB2 receptor

**Figure 2:**
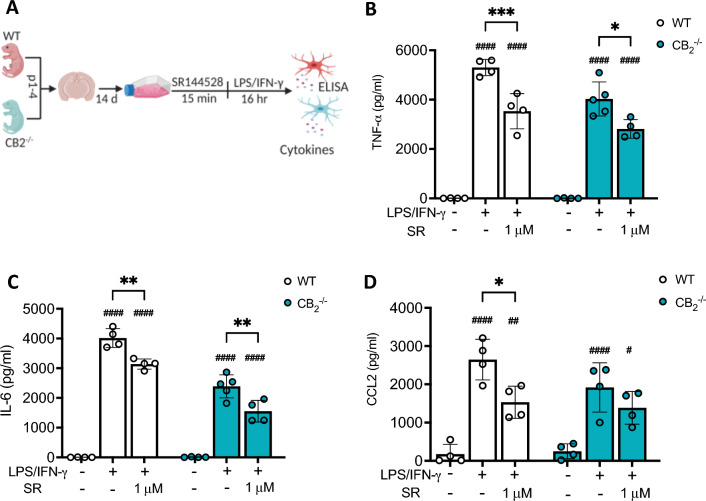
1 µM of SR144528 reduces LPS/IFN-γ-induced microglial inflammatory cytokine secretion independent of the CB2 receptor. (**A**) Experimental setup. Primary microglia of WT and CB2^-/-^ mice were pre-treated with 1 µM SR144528 for 15 min, followed by LPS/IFN-γ stimulation for 16 h. (**B**–**D**) Levels of cytokines/ chemokines were quantified in supernatants using ELISA. (B) TNF-α, (**C**) IL-6, and (**D**) CCL2. N = 4–5 samples/genotype/stimulation (representative data from two independent microglia preparations). Data are presented as mean ± SD. Two-way ANOVA followed by Tukey's multiple comparisons. #### *p* < 0.0001, ## *p* < 0.01, # *p* < 0.05 indicate significance to the untreated control group. Significant differences between LPS/IFN-γ vs. LPS/IFN-γ pre-treated with SR144528 are indicated with *** *p* < 0.001, ** *p* < 0.01, * *p* < 0.05.

To ascertain that the suppression of inflammatory cytokine release in LPS/IFN-γ-stimulated microglia by 1 µM SR144528 was indeed CB2-independent, we treated WT and CB2^-/-^ microglia with 1 µM SR144528 followed by LPS/IFN-γ-stimulation (Fig. 2). We found that this concentration of 1 µM SR144528 significantly reduced secretion of TNF-α (p_(WT)_ = 0.0005, p_(CB2_^-/-^ _)_= 0.0115) and IL-6 (p_(WT)_ = 0.0024, p_(CB2_^-/-^_)_ = 0.0023) in stimulated WT and also CB2^-/-^ microglia (Fig. 2B–C). The reduction in CCL2 secretion did not reach statistical significance in CB2^-/-^ microglia (p_(WT)_ = 0.0218, p_(CB2_^-/-^_)_ = 0.5363), likely due to their generally lower CCL2 secretion capacity (Fig. 2D). These data further confirm our assumption that high concentrations of the CB2 antagonist SR144528 reduced inflammatory cytokine production following LPS/IFN-γ stimulation through an off-target mechanism.

### SR144528 (1 µM) does not affect cell viability in LPS/IFN-γ-stimulated microglia

**Figure 3:**
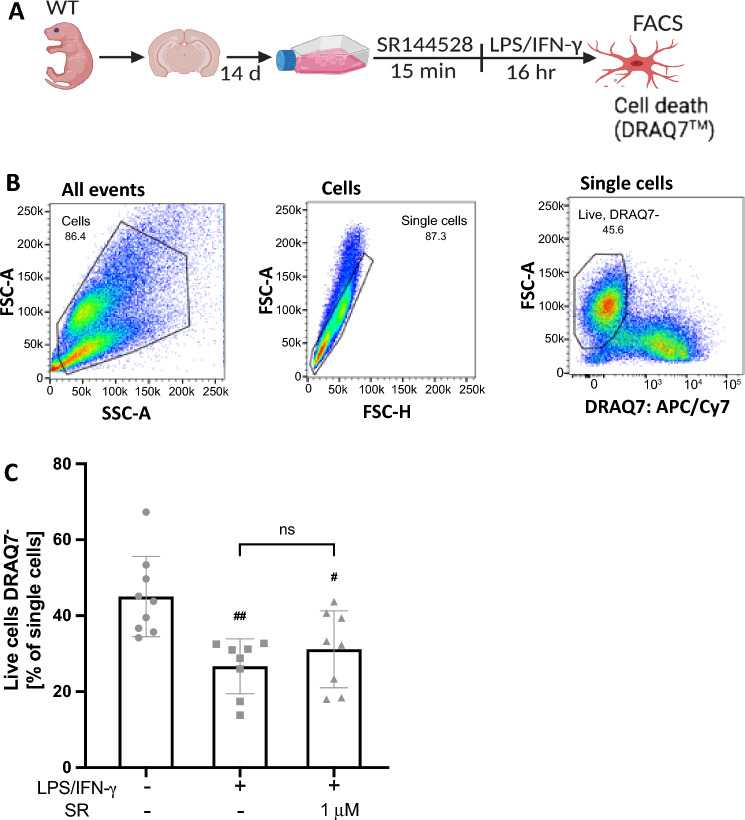
1 µM of SR144528 does not affect the percentage of live cells in LPS/IFN-γ stimulated microglia. (**A**) Experimental setup. Primary microglia were pre-treated with 1 µM SR144528 for 15 min, followed by LPS/IFN-γ stimulation for 16 h. DRAQ7™ was used to stain dead cells (**B**) Gating strategy used for the stained microglia. (**C**) Quantification of the percentage of live microglia cells (DRAQ7 negative). N = 6–9 samples/stimulation (from at least two independent microglia preparations). Data are presented as mean ± SD. One-way ANOVA followed by Tukey's multiple comparisons. ## *p* < 0.01, # *p* < 0.01 indicate significance to the untreated control group.

To test whether the reduced levels of cytokines measured after treatment with 1 µM SR144528 was due to an effect on cell viability, we quantified cell death in primary microglia using flow cytometry (Fig. 3). We found a significant reduction in the percentage of live cells after LPS/IFN-γ stimulation (*p* = 0.0019), which was not affected by pre-treatment with 1 µM SR144528 (Fig. 3). This data indicates that the effect of 1 µM SR144528 was not due to cytotoxicity, thus supporting the finding of potential immune-modulatory off-target effects at micromolar concentrations.

### SR144528 does not affect LPS/IFN-γ-induced microglial Iba1 and CD68 intensities in OHSCs

**Figure 4:**
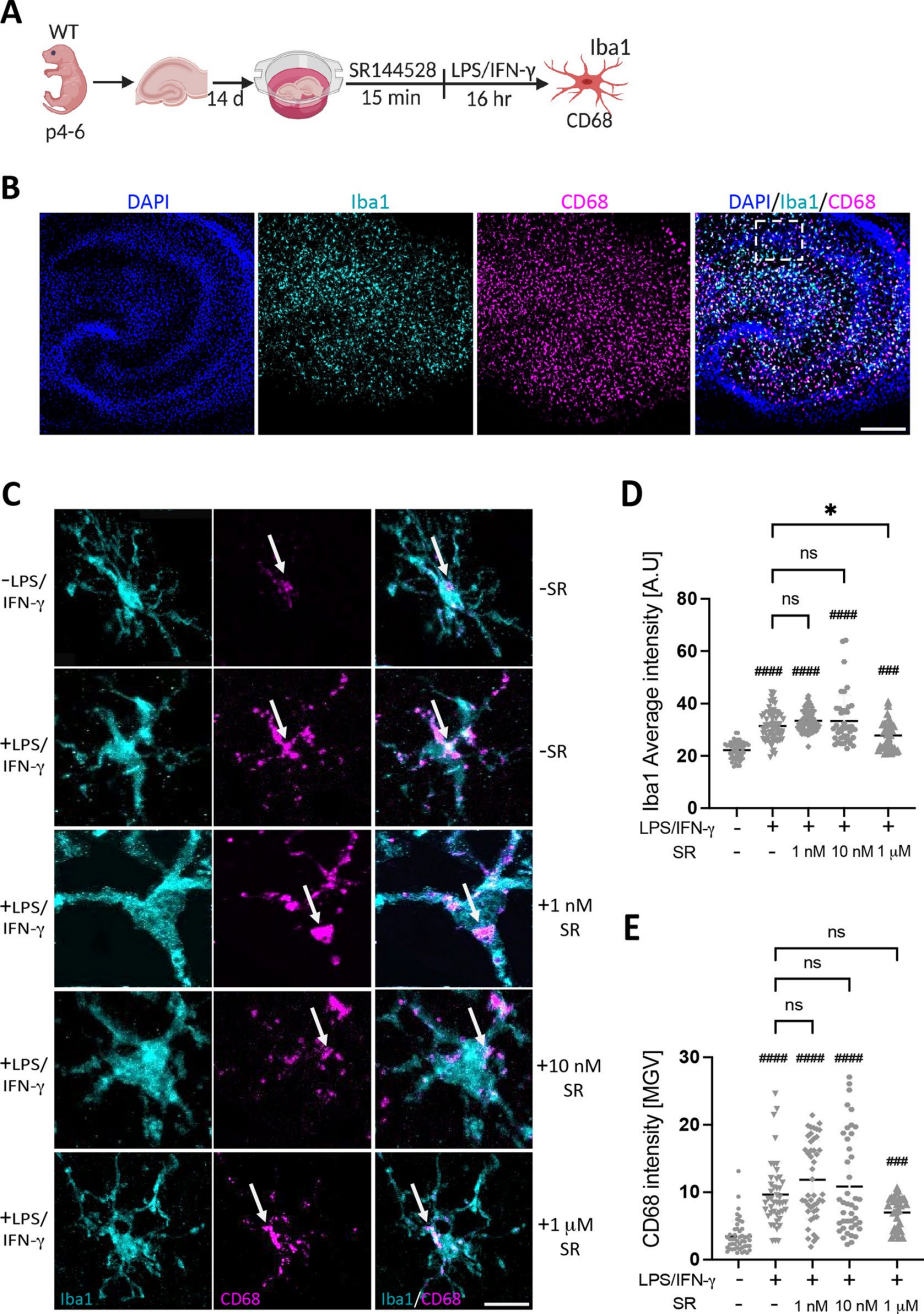
SR144528 does not affect microglial CD68 and Iba1 intensities in LPS/IFN-γ-stimulated OHSCs. (**A**) Experimental setup. OHSCs were pre-treated with SR144528 at the indicated concentrations for 15 min, followed by LPS/IFN-γ stimulation for 16 h. After stimulation, OHSCs were stained for microglial markers and imaged using confocal microscopy. (**B**) Representative images of stimulated OHSCs showing DAPI (blue), Iba1 (cyan), and CD68 (magenta), scale bar = 100 µm, 10× magnification. The white dotted box indicates the region from which representative microglia shown in panel C were obtained. (**C**) Representative images from CA1 pyramidal microglia showing Iba1 and CD68 staining at 40× magnification, scale bar = 30 µm. Quantification of (**D**) Iba1 and (**E**) CD68 intensities. N ≥ 40 microglial cells/stimulation (representative data from two independent OHSCs preparations). Data are presented as mean ± SD. One-way ANOVA followed by Tukey's multiple comparisons was used for normally distributed data, while Kruskal–Wallis test followed by Dunn's multiple comparisons tests was used for data that were not normally distributed. #### *p* < 0.0001, ### *p* < 0.001 indicate significance to the untreated control group. Significant difference between LPS/IFN-γ vs. LPS/IFN-γ pre-treated with SR144528 is indicated with * *p* < 0.05.

We also investigated another endpoint of LPS/IFN-γ stimulation, namely the microglial marker Iba1 (ionized calcium-binding adapter molecule 1) and the lysosomal marker and indicator for phagocytosis CD68, in activated microglia. For this purpose, we used organotypic hippocampal slice cultures (OHSCs), a model that supports the physiological maintenance of all hippocampal cell types in culture. We pre-treated OHSCs with SR144528 (1, 10 nM and 1 µM), followed by LPS/IFN-γ stimulation for 16 hr (Fig. 4) and analyzed Iba1 and CD68 staining intensities within microglia in the CA1 pyramidal layer of the treated OHSCs (Fig. 4B–C). Our result showed that both Iba1 and CD68 intensities were significantly increased after LPS/IFN-γ stimulation. However, pre-treatment with SR144528 did not considerably change Iba1 or CD68 intensities at 1 nM and 10 nM (Fig. 4D–E). Here, 1 µM of SR144528 reduced Iba1 but not CD68 intensity in LPS/IFN-γ stimulated OHSCs (Fig. 4D–E). This data additionally supports that blockade of CB2 signaling by SR144528 at nanomolar concentrations does not influence microglial activity after LPS/IFN-γ stimulation.

### SR144528 does not considerably alter LPS/IFN-γ-induced activated microglial morphology in OHSCs

**Figure 5:**
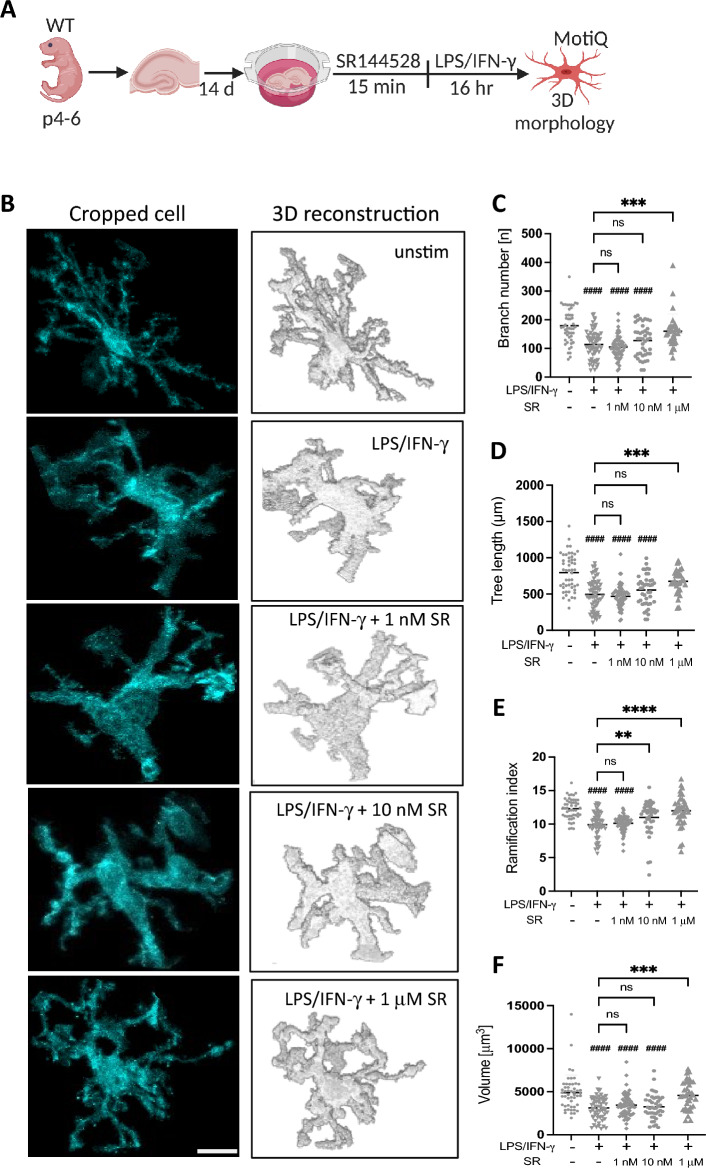
SR144528 does not alter 3D microglial morphology in LPS/IFN-γ stimulated OHSCs. (**A**) Experimental setup. OHSCs were pre-treated with SR144528 at the indicated concentrations for 15 min, followed by LPS/IFN-γ stimulation for 16 h. After stimulation, OHSCs were stained for microglial markers and imaged using confocal microscopy. Microglia morphology was analyzed based on Iba1 staining using MotiQ. (**B**) Representative images of cropped and 3D reconstructed microglia, scale bar = 30 µm. (**C**–**F**) Quantification of morphological parameters of reconstructed microglia. (**C**) Branch number, (**D**) Tree length, (**E**) Ramification index, (**F**) Volume. N ≥ 40 microglial cells/stimulation (representative data from two independent OHSCs preparations). Data are presented as mean ± SD. One-way ANOVA followed by Tukey's multiple comparisons was used for normally distributed data, while Kruskal–Wallis test followed by Dunn's multiple comparisons tests was used for data that were not normally distributed. #### *p* < 0.0001 indicates significance to the untreated control group. Significant differences between LPS/IFN-γ vs. LPS/IFN-γ pre-treated with SR144528 are indicated with **** *p* < 0.0001, *** *p* < 0.001, ** *p* < 0.01.

Finally, we determined microglial morphology using the MotiQ plugin^20^ to characterize 3D morphometric parameters of microglia in LPS/IFN-γ-stimulated OHSCs pre-treated with SR144528 (Fig. 5B). We observed that LPS/IFN-γ-stimulated microglia exhibited ameboid-like features such as fewer branches (Fig. 5C), shorter tree lengths (Fig. 5D), and lower cell volume (Fig. 5F). These parameters were not altered by SR144528 (1 and 10 nM). In contrast, the ramification index- a measure of the overall complexity of cell shape was significantly increased with 10 nM of SR144528 pre-treatment (Fig. 5E), whereas all the parameters tested were significantly reduced with 1 µM of SR144528 pre-treatment (Fig. 5C–F). This finding further indicates that SR144528 at nanomolar concentrations does not generally influence the LPS/IFN-γ-induced ameboid-like microglial morphology, which is a common feature of activated microglia.

## Discussion

In this study, we asked if acute pharmacological inhibition of the CB2 receptor mimics the anti-inflammatory effect observed in constitutive CB2 deletion (CB2^−/−^) following stimulation with LPS/IFN-γ. Our results show that the blockade of CB2 signaling with the CB2 antagonist, SR144528 did not recapitulate the result of CB2^−/−^ with respect to modulating LPS/IFN-γ-induced microglial activation. It is likely that the constitutive lack of CB2 receptors affects microglia development or functions, making them less responsive to inflammatory stimuli. In addition, our data emphasize the potential off-target effects of SR144528 at micromolar concentrations.

We analyzed several aspects of microglial activation in primary microglia and OHSCs pre-treated with SR144528 and stimulated with LPS/IFN-γ. SR144528 is the most widely-used CB2 antagonist for pharmacological studies due to its high potency and selectivity for both mouse and human CB2 receptors^18,21^. It has been employed in numerous studies, often to block the effects of CB2 agonists^9,11,22^. The SR144528 concentration required to produce its half maximum inhibition (Ki)^18^ is 0.6 nM. This compound thus blocks CB2 receptors at low nanomolar concentrations. We showed that pre-treatment with SR144528 at 1 nM and 10 nM concentrations did not affect the secretion of inflammatory cytokines IL-6, TNF-α, and CCL2 in LPS/IFN-γ-stimulated microglia. Furthermore, SR144528 at 1 nM and 10 nM did not alter staining intensities of pro-inflammatory microglial activation markers, Iba1, which is often increased during microglia activation^14,23^ and CD68, which is also enhanced during inflammation^10^. Additionally, we assessed the morphological changes of microglia resulting from LPS/IFN-γ stimulation in a 3D reconstruction analysis. It is of common notion that the shape of microglia gives helpful information about their activation state^14,23–25^. After LPS/IFN-γ stimulation of OHSCs, we observed activated morphological changes characterized by fewer microglial branch numbers, shorter tree lengths, reduced ramification, and cell volume in the stimulated OHSCs. Pre-treatment with SR144528 at 1 and 10 nM failed to produce a difference in all but one of these parameters, supporting its inability to generally prevent LPS/IFN-γ-induced microglial activation. Only the ramification index was increased by 10 nM SR144528, indicating that either CB2 signaling has a distinct influence on this morphology parameter or reflects a particularly sensitive off-target effect. Together, these data strongly suggest that acute inhibition of CB2 signaling does not modulate microglial responses to LPS/IFN-γ, which is in contrast with our previous results^14^ showing that the genetic deletion of CB2 suppressed LPS/IFN-γ-mediated microglial activation.

It is noteworthy that a high concentration of 1 µM SR144528 suppressed LPS/IFN-γ-induced microglial cytokine secretion, Iba1 intensity and morphological alterations, an effect that was not dependent on the CB2 receptor. This finding is relevant, because many studies in microglia or macrophages used SR144528 at 1 µM or higher concentrations to reverse the effect of CB2 agonists in inflammatory settings^9,11,22,26^. Even though SR14428 is an excellent CB2-specific antagonist, some off-target effects have been described at micromolar concentrations^21^. Considering that 1 µM is more than a thousand-fold greater than its Ki, it is likely that SR144528 had CB2-independent off-target immune-modulatory functions. Indeed, data from CB2 ligand profiling revealed that SR144528 has off-target activities on other proteins, namely adenosine A3 receptor (A_3_AR) and phosphodiesterase 5 (PDE5)^21^. Another study showed that SR144528 and AM-251 are inhibitors of the enzyme acyl CoA: cholesterol acyltransferase (ACAT)^27^. While it was beyond the scope of our present study to identify the CB2-independent mechanisms of 1 µM SR144528 in microglia, these off-targets might be involved. One study demonstrated a pro-inflammatory effect of the A_3_AR agonist, CI-IB-MECA, on LPS-induced TNF-α secretion in peritoneal macrophages^28^. Another study showed that inhibiting ACAT1 in macrophages overloaded with cholesterol produced a lower inflammatory profile^29^.

Where genetic knockout models are important tools for understanding specific roles of genes and their underlying mechanisms in neuropathological or inflammatory conditions^30–33^, the permanent genetic alteration can initiate adaptive developmental changes^34^. It can thus be difficult to distinguish between direct effects due to the targeted gene deletion and indirect effects, requiring other means of functional gene or protein invalidation, such as pharmacological agonists and antagonists^35^. Here, we found that acute SR144528 treatment did not affect LPS/IFN-γ-induced microglial activation, whereas constitutive deletion of CB2 reduced TLR-induced microglial activation in our previous study^14^, suggesting that the latter is not caused by an acute lack of CB2 signaling during stimulation. Instead, these findings indicate that CB2^−/−^ microglia are primed differently, making them somewhat resistant to LPS/IFN-γ stimulation. A few studies have reported discrepancies between genetic deletion and pharmacological inhibition of proteins^17,35^. For example, differential effects of soluble epoxide hydrolase inhibition and genetic deletion was shown on cardiac fibrosis^17^ while another study demonstrated that the lysophosphatidate LPA_1_ receptor antagonist Ki16425 did not totally mimic the effects of the constitutive lack of LPA1 receptors on depression-like behavioral tests in mice^35^.

In our previous studies, we reported that primary microglia from CB2^−/−^ mice showed decreased secretion of inflammatory cytokines and decreased surface expression of activation markers following LPS/IFN-γ stimulation^6^. Recently, we revealed that the lack of CB2 attenuated TLR-induced microglial activation^14^. These findings suggested that the CB2^−/−^ mice are less prone to inflammatory stimulation of microglia. Somewhat contradictory to these observations, CB2 activation with CB2 agonists also exhibited anti-inflammatory effects on microglia and macrophages in response to inflammatory stimuli. For example, the CB2 agonists JWH-015 and AM1241 were shown to dampen inflammatory responses to IFN-γ or LPS/IFN-γ stimulation in primary microglia and N9 microglia respectively^7,15^. Additionally, JWH-133 was reported to increase IL-10 secretion in LPS/IFN-γ-stimulated macrophages, which was reversed by SR144528^9^. Based on these findings, it is evident that pharmacological activation/inactivation of CB2 and genetic manipulations can have different outcomes. Our results are most likely due to compensatory mechanisms, as we have previously reported about 200 genes (including gene ontology terms for response to bacteria and viruses) that are differentially expressed between unstimulated WT and CB2^−/−^ microglia^14^. Some of these genetic changes probably make CB2^−/−^ microglia less sensitive to inflammatory stimulation.

## Methods

### Mice

C57BL/6J (WT) and B6.cg-Cnr2tm1Zim (CB2^−/−^)^30^ mice were used in this study. The C57BL/6J mice were originally procured from a Charles River commercial breeder and bred in-house. The B6.cg-Cnr2tm1Zim mice were bred homozygously and backcrossed to C57BL/6J line every six generations to reduce the chance of genetic drift. All animals were housed in the animal facility unit of the University of Bonn under specific pathogen-free conditions. They were subjected to a 12-h dark/light cycle with ad libitum access to food and water. Animal care and organ retrievals were performed according to the guidelines of the European Communities Directive 86/609/E.E.C. and the German Animal Protection Law regulating animal research and were approved by the Landesamt für Natur-, Umwelt-, und Verbraucherschutz (LANUV NRW), Germany (01_Organentnahme). The study was conducted in accordance with ARRIVE guidelines.

### Primary microglia culture

Primary neonatal microglia were prepared as previously described by Reusch et al.^14^ with few modifications. Briefly, brains were extracted from decapitated WT or CB2^−/−^ pups (p1 to p4) and transferred into ice-cold HBSS (Gibco, cat# 14175-053). Meninges were gently removed, and cortices dissociated to reach a single-cell suspension. Resuspended cells were sieved through a 70 µm cell strainer (Falcon, cat# 352350) and cultivated in high glucose DMEM + GlutaMAX (Gibco, cat# 61965-026) supplemented with 1% penicillin/streptomycin (Gibco, cat# 15140-122) and 10% heat-inactivated fetal bovine serum (PAA, cat# A15-108) in a poly-L-Lysine coated T75 culture flask (Greiner bio-one, cat# 658175). The medium was changed twice a week until confluency was reached (approximately 14 days). Microglia were harvested by shaking method. The flasks were placed on a shaker set at 200 rpm for 1 h and reseeded in 12-well plates (Greiner bio-one cat# 665180) at a density of 1.5 × 10^5^ cells/ml.

### Organotypic hippocampal slice cultures

Organotypic hippocampal slice cultures (OHSCs) were prepared as previously described^14^. Briefly, brains were extracted from decapitated WT pups (p4 –p6) and transferred into ice-cold dissection medium composed of minimum essential medium ((MEM) Gibco, cat# 11090-081), supplemented with 1% HEPES (Gibco, cat# 15630-056), 1% penicillin/streptomycin (Gibco, cat# 15140-122), and 1% glutamine (Gibco, cat# 25030-024). Cerebelli were removed, and 2–3 brains (dorsal side up) were gently glued to the vibratome specimen plate (Leica VT1200 S, cat# 048142066R0901) supported by a 2% agarose block. The resulting specimen platform containing the glued brains was immersed in the vibratome buffer tray containing an ice-cold dissection medium. 350 µm thick slices were sectioned horizontally and rapidly transferred to a fresh dissection medium. The hippocampal slices with intact CA1, CA2, CA3, and dentate gyrus were dissected and placed on 0.4 µm semi-porous membrane inserts (Merck Millipore Ltd, cat# PICMORG50). The membrane inserts with 3 slices per insert were further transferred into 6-well plates containing 1 ml fresh culture medium per well. The culture medium was composed of MEM containing 25% Horse serum (Gibco, cat# 26050-088), 25% HBSS (cat# 24020-091), 1% glutamine, 1% penicillin/streptomycin, 4.5% D-Glucose (Sigma cat# G8769) and 1% Amphotericin B (Sigma cat# A2942). The hippocampal slices were cultured in an incubator set at 37 °C and 5% CO_2_. The medium change occurred every other day for 14 days.

### Cell treatment with LPS/IFN-γ and SR144528

15 min before LPS/IFN-γ stimulation, microglia or OHSCs were first pre-treated with SR144528 (Sigma, cat# SML1899) dissolved in DMSO (AppliChem GmbH, cat# A3672,0250) at various concentrations (1, 10, 100 nM, 0.5 and 1 µM), after which they were stimulated with 100 ng/ml Lipopolysaccharide; LPS (Sigma-Aldrich, cat# L4516-1MG) and 20 ng/ml interferon-gamma; IFN-γ (R&D Systems, cat# 485-MI) for 16 h. Schematic representation of experimental setup was created in BioRender.com.

### ELISA

The levels of murine CC-chemokine ligand 2 (CCL2), interleukin 6 (IL-6), and tumor necrosis factor (TNF-α) were quantified in microglia cell culture supernatants using the uncoated Thermo Fisher ELISA kits (cat# 88-7064-88, cat# 88-7391-88 and cat# 88-7324-88 respectively). The assay procedures were carried out following the manufacturer's manual. Absorbance was read at 450 nm using a Dynex Technologies microplate reader.

#### Flow cytometry

Stimulated microglia were harvested in 2 mM EDTA in PBS and blocked with Fc-receptor blocker (CD16/32 antibody (Biozol, cat# 101302)) diluted 1:200 in FACS buffer (PBS + 2% FCS). Dead cells were stained with DRAQ7™, a far-red emitting nuclear stain that is non-permeant to live cells. Samples were measured using a FACS Canto II (BD Biosciences) and data was analyzed using FlowJo version 10.0.7 (Tree Star Inc).

### Immunohistochemistry

OHSCs were stained as free-floating sections attached to the membrane inserts. Slices were washed thrice in ice-cold PBS and then fixed in 4% paraformaldehyde (PFA) solution for 4 h at room temperature (RT). Fixed slices were permeabilized in 0.5% Triton X100 and 0.1% 1 M sodium azide (NaN_3_) in PBS for 72 h at 4 °C. After washing thrice in PBS, slices were blocked for 48 h at 4 °C in 0.5% Triton X100, 10% normal goat serum, and 0.1% NaN_3_ in PBS. Blocked slices were stained for 72 h at 4 °C with 1:1000 primary antibodies (rabbit anti-Iba1 (AB_839504) and rat anti-CD68 (AB_322219)). After washing, slices were incubated with secondary antibodies, goat-anti-rat AF488 (AB_2534074) and goat-anti-rabbit AF467 (AB_2535813) for 4 h at room temperature. Slices were subsequently mounted on glass slides using DAPI Fluoromount-G^(R)^ (SouthernBiotech, cat# 0100-20).

### Confocal microscopy and image acquisition

High-resolution images were acquired using a Leica TCS SP8 confocal laser scanning microscope (S/N 8100000359) with a 40× water immersion objective (NA = 1.1, pinhole = 1 A.U.). In each independent experiment, 10 z-stacks (with 0.5 µm step size, resolution 1024 × 1024 px, 0.1 µm/px) were acquired per stimulation from the strata pyramidal and radiatum area of the CA1 hippocampal region. Imaging and analysis were performed by an investigator blinded to the stimulation.

### Microglial Image analysis and 3D reconstruction

Microglial confocal images were quantified using ImageJ (Fiji version 2.3.0/1.53q). Microglial morphology was quantified using the MotiQ toolbox designed to analyze and reconstruct microglia as described by Hansen et al.^20^. Briefly, 40 or more microglial cells (Iba1^+^ cells^14^) were analysed per stimulation. The resulting cropped-out single-cell images were segmented using the MotiQ thresholder (selected algorithm: "Huang"). The thresholded images were further analyzed using the MotiQ 3D analyzer plugin. The particle filter option was set to 0.5 µm voxel depth and 10,000 voxels minimum particle volume. The Iba1 mean intensity of the microglia cells was determined as the average intensity of all the voxels in the original image that was positive in the segmented, particle-filtered images. The 3D reconstruction parameters, branch number, ramification index, tree length, and volume were automatically generated using the ImageJ MotiQ plugin "Skeletonize3D" and analyzed using the ImageJ MotiQ plugin "Analyze Skeleton"^20^.

### CD68 intensity

CD68 intensity was measured within each Iba1 + microglia cell using ImageJ. Briefly, using the Z project command, the maximum Z-projections of Iba1 (Ib MAX) and CD68 (CD MAX) were generated from the respective confocal Z stack images. Next, microglia cells were manually selected from the Ib MAX and saved as Iba1 region of interest (ROI). The resulting CD MAX images were thresholded using the threshold command and signal intensity (mean gray value) was measured within the Iba1 ROI using the measure command.

### Statistical analysis

The data shown are representative of at least three independent experiments for primary microglia and two independent experiments for OHSCs. Results are presented as mean ± SD. Statistical analysis was performed using GraphPad Prism Version 9.0. D’Agostino-Pearson omnibus test was used to determine if the data were normally distributed for group sizes n > 40. Data were analyzed using one-way or two-way analysis of variance (ANOVA), followed by Tukey's post hoc test for multiple comparisons between groups. In the case that more than one group was not normally distributed, Kruskal–Wallis test followed by Dunn's multiple comparisons test was used. *P* values  < 0.05 were considered statistically significant.

## Data Availability

Datasets from this study are available upon request. The raw data supporting our study conclusions will be made available by the corresponding author—Andreas Zimmer, to any qualified researcher.
