# Author Correction: Pharmacological blockade of cannabinoid receptor 2 signaling does not affect LPS/IFN-γ-induced microglial activation

**DOI:** 10.1038/s41598-023-42771-1

**Published:** 2023-09-21

**Authors:** Bolanle Fatimat Olabiyi, Anne-Caroline Schmoele, Eva Carolina Beins, Andreas Zimmer

**Affiliations:** 1 Institute of Molecular Psychiatry, Medical Faculty, University of Bonn, Bonn, Germany; 2 Institute of Human Genetics, University of Bonn, School of Medicine & University Hospital Bonn, Bonn, Germany

Correction to: *Scientific Reports* 10.1038/s41598-023-37702-z, published online 10 July 2023

The original version of this Article contained an error in Figure 4, where some arrows in Panel C appeared displaced. The original Figure 4 and accompanying legend appear below.

**Figure 4:**
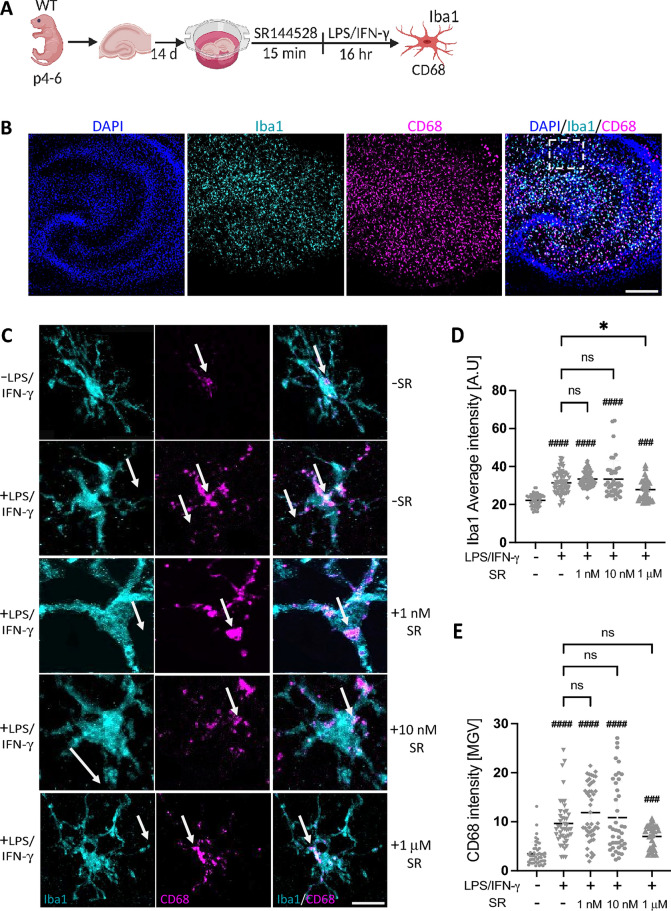
SR144528 does not affect microglial CD68 and Iba1 intensities in LPS/IFN-γ-stimulated OHSCs. (A) Experimental setup. OHSCs were pre-treated with SR144528 at the indicated concentrations for 15 min, followed by LPS/IFN-γ stimulation for 16 h. After stimulation, OHSCs were stained for microglial markers and imaged using confocal microscopy. (B) Representative images of stimulated OHSCs showing DAPI (blue), Iba1 (cyan), and CD68 (magenta), scale bar = 100 µm, 10× magnification. The white dotted box indicates the region from which representative microglia shown in panel C were obtained. (C) Representative images from CA1 pyramidal microglia showing Iba1 and CD68 staining at 40× magnification, scale bar = 30 µm. Quantification of (D) Iba1 and (E) CD68 intensities. N ≥ 40 microglial cells/stimulation (representative data from two independent OHSCs preparations). Data are presented as mean ± SD. One-way ANOVA followed by Tukey's multiple comparisons was used for normally distributed data, while Kruskal–Wallis test followed by Dunn's multiple comparisons tests was used for data that were not normally distributed. ####*p* < 0.0001, ###*p* < 0.001 indicate significance to the untreated control group. Significant difference between LPS/IFN-γ vs. LPS/IFN-γ pre-treated with SR144528 is indicated with *p < 0.05.

The original Article has been corrected.

